# REDUCING OR STOPPING MEDICATIONS FOR CHRONIC PAIN: VIEWS AMONG COMMUNITY-DWELLING OLDER ADULTS

**DOI:** 10.1093/geroni/igae098.3022

**Published:** 2024-12-31

**Authors:** Hyunjin Noh, Haelim Jeong, Denise Kan, Lewis Lee

**Affiliations:** University of Alabama, Tuscaloosa, Alabama, United States; University of Alabama, Tuscaloosa, Alabama, United States; University of Alabama, Tuscaloosa, Alabama, United States; University of Alabama, Tuscaloosa, Alabama, United States

## Abstract

Effective pain control is vital in enhancing quality of life. As the dangers of adverse drug interactions are recognized, there is a shift towards reducing reliance on medications that could be ineffective or harmful (known as deprescribing) and a push for embracing non-drug methods in managing pain. However, limited research exists on how older adults perceive deprescribing. Therefore, this study aimed to understand the views of deprescribing among community-dwelling older adults living with multiple health conditions and chronic pain. Participants were recruited from the Alabama Area Agency on Aging offices. Eligible participants were 65+, Alabama residents living outside of nursing homes, cognitively intact, have more than one chronic illness and chronic pain, and take medications for the illnesses, including pain medicine. Individual, open-ended interviews were conducted by phone, asking about their experiences with taking multiple medications and thoughts about deprescribing. Thematic analysis of interviews with 30 participants revealed facilitators, barriers, and needs for deprescribing. Facilitators for deprescribing included interest in more holistic methods for pain management, concerns for long-term side effects, and desire for better quality of life, while barriers included fear of worsening pain, reluctance to change in medical regimen, and lack of support from healthcare providers. Needs for deprescribing included credible information from their healthcare providers about deprescribing and social support in deprescribing decision-making. These findings suggest that older adults may benefit from education and advocacy efforts designed to improve their knowledge and self-efficacy and healthcare providers’ willingness to openly communicate with older adults about deprescribing.

